# Hemangioma of the Tongue: A Case Report

**DOI:** 10.7759/cureus.67044

**Published:** 2024-08-16

**Authors:** Shubhani Kharkate, Swapnil Mohod, Monal M Kukde, Aakanksha V Tiwari, Samiksha A Bute

**Affiliations:** 1 Dentistry, Sharad Pawar Dental College and Hospital, Datta Meghe Institute of Higher Education and Research, Wardha, IND; 2 Dentistry, Dr. Panjabrao Alias Bhausaheb Deshmukh Memorial Medical College, Amravati, Amravati, IND; 3 Dentistry, Datta Meghe Medical College and Shalinitai Meghe Hospital and Research Center, Nagpur, IND; 4 Oral Medicine and Radiology, Sharad Pawar Dental College and Hospital, Datta Meghe Institute of Higher Education and Research, Wardha, IND

**Keywords:** oral cavity, swelling, hemangioma, benign tumors, surgery

## Abstract

Vascular anomalies include a wide range of tumors and malformations. Hemangioma is the most frequent vascular defect. Hemangiomas are benign endothelial cell tumors most frequently detected in children but uncommon in adults. Most of them affect the neck and head regions but rarely involve the palate, lips, tongue, and buccal mucosa. Treatment for oral hemangiomas should begin as soon as possible because they are clinically significant. Patients are at an increased risk of developing tongue hemangiomas due to the tongue's high flexibility and muscular structure, which makes it more vulnerable to trauma and its effects. They grow and proliferate within a few weeks of birth, with the majority of their components undergoing involution. Females are more likely than males to develop hemangiomas. Hemangiomas are treated with surgery, laser therapy, radiofrequency, sclerosing agents, radiation treatment, cryosurgery, corticosteroids, embolization, electrocauterization, and interferon. When assessing treatment options for hemangiomas, it is crucial to consider various criteria, such as the lesion's hemodynamics, the patient's age, and the location, size, and feasibility of the planned procedure. This report describes a case of a 19-year-old female diagnosed with a hemangioma located on the middle third of the dorsal aspect of the right lateral border of the tongue.

## Introduction

Based on the cellular approach, Mulliken and Glowacki classified vascular lesions into two distinct types: hemangioma and vascular malformation [1]. The hemangioma type is characterized by endothelial proliferation, which grows quickly before gradual involution. In general, 90% of cases resolve on their own by age 12, whereas 5% to 10% affect infants under one year old [2]. The vascular malformation type, which affects between 0.3% and 1% of newborns, is present from birth and does not show signs of endothelial growth. Hemangiomas are benign proliferative lesions that closely resemble normal vessels in the vascular tissue of the arteries. These lesions have multiple endothelial cells necessary for lining the lumen. Although they are rare in the oral and perioral regions, they are comparatively more prevalent in the head and neck regions. Intraoral infections can occur in the buccal mucosa, palatal mucosa, gingiva, tongue, and lips [3]. However, hemangiomas have been observed more frequently in the lower lip, lateral border of the tongue, and buccal mucosa; thus, they can occur in any part of the oral cavity or pharyngeal location [4]. Hemangiomas often form two to four weeks after birth and grow quickly until six to eight months of age, at which point they begin to slow down. In 70% of instances, they regress spontaneously and begin to involute by the time they are five to eight years old [5]. Hemangiomas are more commonly observed in females, White children, twins, premature newborns, and children born to older mothers.

Hemangiomas present clinically as a swelling, nodule, macule, papule, or tumor and can range in size from a few millimeters (mm) to several centimeters (cm). Due to the involvement of blood in the lesion, it is often red or bluish-purple in most cases. Depending on the quantity of connective tissue and its location - deep or superficial - the consistency may be fibrous or elastic. Superficial lobulated hemangiomas blanch in response to pressure, while deeper lesions are often dome-shaped, seldom blanch, and have a normal or blue surface color. Oral pedunculated hemangiomas are extremely uncommon.

Hemangiomas are classified in various ways, such as capillary, cavernous, or miscellaneous, including verrucous, venous, and arteriovenous hemangiomas, according to Enzinger and Weiss [6]. Large, thin-walled capillaries cause prevalent injuries through infiltration from cavernous hemangiomas. Capillary hemangioma, the most frequent type, is identified by the presence of tiny vessels within connective tissue with a deficiency of elastin, which causes small localized lesions [7]. Among the various locations for head and neck hemangiomas, the tongue demands careful consideration because it is prone to minor injuries that can result in bleeding and ulceration. Breathing issues and difficulty swallowing might also result from it; however, in most situations, cosmetic concerns are the main focus [8,9]. Hemangiomas often regress in response to medical intervention or conservative therapy [10,11]. Hemangiomas are primarily treated with observation; nevertheless, patients sometimes seek more intrusive forms of care due to the unbearable discomfort that results. In certain cases, intralesional sclerosing drugs are advised as a therapy for hemangiomas, and surgical excision is another option [8]. This report describes a case of a 19-year-old patient diagnosed with a hemangioma located on the dorsal middle third of the right lateral border of the tongue.

## Case presentation

**Figure 1:**
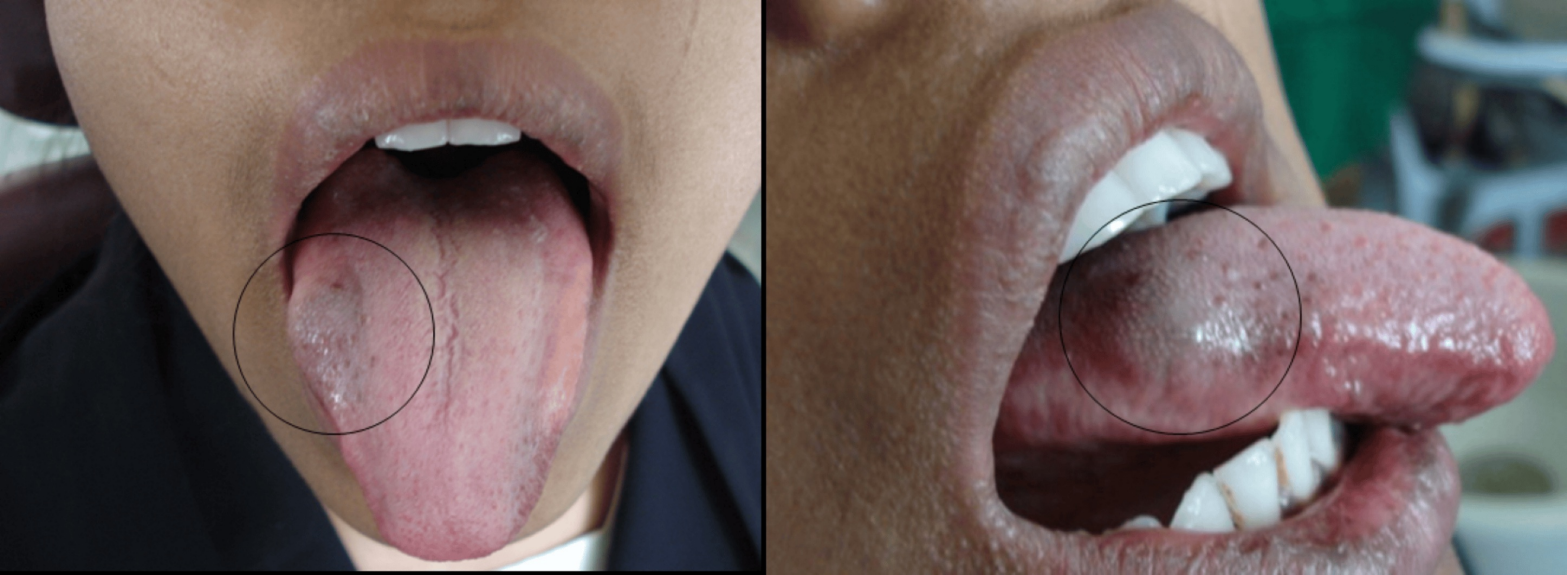
Lesion seen on the right lateral border of the tongue

A 19-year-old girl reported to the Outpatient Department of Oral Medicine and Radiology with a complaint of painless swelling on the dorsal aspect of the tongue for three years. The patient was alright three years back then she had trauma over the the right side of the face and gave a history of tongue bite. The patient first noticed the swelling three years ago. It started initially as a pear-shaped growth (about 0.5 x 1 cm) and developed progressively to its current 1 x 1.5 cm size. The patient has a history of difficulty in chewing and speech. There is no history of bleeding or pus discharged from swelling. There were no relevant dental, medical, or family records to report. On general examination, all vital signs were within normal limits, and the patient's build was normal. On intraoral examination, a solitary blue-colored swelling was present on the middle third of the dorsal aspect of the right side lateral border of the tongue, as shown in Figure 1.

**Figure 2:**
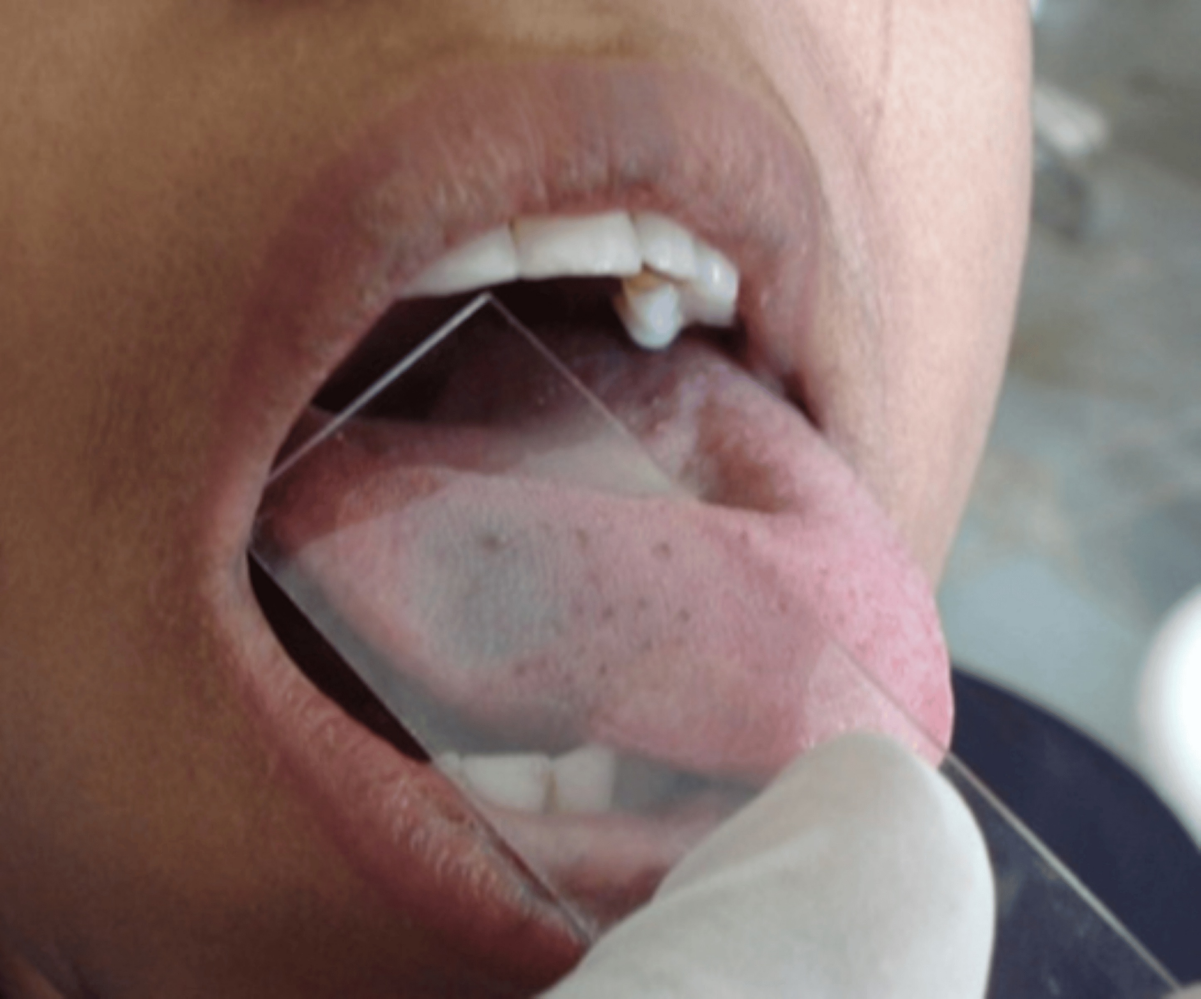
Diascopy test shows no blanching

The swelling was sessile, with a smooth surface and roughly rounded in shape. The margins were diffused. The swelling did not show any bleeding when touched. It is well-circumscribed and soft in consistency upon palpation. On palpation, pulsation was absent. There was no restriction on tongue movement. The diascopy test was done using a glass slab, as shown in Figure 2.

The slide is held onto the lesion with gentle pressure for one to two minutes. The diascopy test showed no blanching when the slide was removed. Based on clinical signs, tongue hemangioma was provisionally diagnosed. Vascular malformation and infantile hemangioma could be other provisional diagnoses. Unlike hemangiomas, vascular malformations are congenital abnormalities involving abnormal development or growth of blood vessels. Vascular malformations are present at birth and persist throughout life unless treated. Infantile hemangioma of the tongue is a benign vascular tumor that appears as an abnormal cluster of blood vessels in the tongue. USG and MRI of the tongue were advised to the patient. Before establishing a definitive treatment plan, additional diagnostic techniques, like color Doppler ultrasound and CT angiography, were advised, but the patient failed to follow up for further treatment.

## Discussion

A hemangioma is defined as "a benign tumor of dilated blood vessels." Salmon patch, strawberry hemangioma, and port-wine stain are some manifestations. It is identified by the hyperplasia of blood vessels in a specific location of the submucosal connective tissue, generally veins and capillaries [12]. Hemangiomas develop with cellular proliferation. Seven percent of these tumors are benign. Typically, 70-90% of them grow during the first one to four weeks [13]. The most prevalent benign vasoformative tumors in children and infants are called hemangiomas. Vasoformative tumors are categorized into three main groups: vascular tumors, vascular malformations, and associated syndromes [14]. Vascular tumors are further classified as malignant (angiosarcoma), locally aggressive (Kaposi sarcoma), and benign (hemangioma).

Based on its histological characteristics, hemangioma can be divided into capillary or cavernous types [15]. Capillary hemangiomas are made up of large numbers of small capillaries, which are set in connective tissue stroma whose thickness is variable and which is lined by one layer of endothelial cells. Cavernous hemangiomas are characterized by extensive, thin-walled vessels known as sinusoids, which are bordered by epithelial cells and partitioned by delicate layers of connective tissue septa [16]. Although hemangiomas are not very frequent in the tongue region, minor injuries to the tongue more often provoke bleeding and ulceration, as well as difficulties in swallowing and breathing. Generally, though, the main concern is cosmetic. The hemangioma is a soft mass; it may be sessile or pedunculated, smooth or lobulated, and it can measure from millimeters to centimeters [17,18]. Hemangiomas can clinically and histopathologically resemble other lesions. Conditions to consider in the differential diagnosis of hemangioma include squamous cell carcinoma, telangiectasia, pyogenic granuloma, and chronic gingival hyperplasia. Pyogenic granulomas, which are reactive lesions that bleed easily, expand rapidly, and are usually accompanied by ulceration and inflammation, are often mistaken for hemangiomas [19].

To identify the involved osseous structures, draining veins, and feeding arteries, MR angiography and CT angiography are necessary. Imaging techniques, such as color Doppler ultrasonography, may also be needed to assist in diagnosing the lesion and determining the type of blood flow [20]. Various therapy methods have been used to slow the growth and accelerate the regression of hemangiomas. Based on size and location, treatment options for smaller and peripheral lesions include sodium tetradecyl sulfate, hypertonic glucose solution, and sodium sclerotherapy [21]. Other options include radiation, electrocoagulation, laser treatment, cryotherapy, and conventional surgical excision. Treatments for larger or intraosseous lesions, where aesthetics are important, should involve embolization or obliteration of the lesion and surrounding arteries. Therefore, it is essential to achieve the lesion's involution to prepare it for a future surgical procedure [22].

## Conclusions

One of the most frequent tumors seen in newborns and children is hemangioma. Although hemangiomas are benign endothelial cell tumors commonly seen in the head and neck regions, they are relatively infrequent in the oral cavity. Determining the clinical behavior of the tumor and its potential consequences requires early diagnosis and biopsy. The location, size, and age of the lesion all influence the treatment options. Most congenital hemangiomas resolve on their own without medical intervention. The prognosis and diagnosis of the particular vascular abnormality should be taken into consideration while planning the treatment approach.
